# Deformation velocity imaging using optical coherence tomography and its applications to the cornea

**DOI:** 10.1364/BOE.8.005579

**Published:** 2017-11-13

**Authors:** Samuel Lawman, Peter W. Madden, Vito Romano, Yue Dong, Sharon Mason, Bryan M. Williams, Stephen B. Kaye, Colin E. Willoughby, Simon P. Harding, Yao-Chun Shen, Yalin Zheng

**Affiliations:** 1 Department of Electrical Engineering and Electronics, University of Liverpool, Liverpool, L69 3GJ, UK; 2 Department of Eye and Vision Science, Institute of Ageing and Chronic Disease, University of Liverpool, Liverpool, L7 8TX, UK; 3 St. Paul's Eye Unit, Royal Liverpool University Hospital, Liverpool L7 8XP, UK

**Keywords:** (110.4500) Optical coherence tomography, (170.4500) Optical coherence tomography, (120.5050) Phase measurement, (170.6935) Tissue characterization

## Abstract

Optical coherence tomography (OCT) can monitor human donor corneas non-invasively during the de-swelling process following storage for corneal transplantation, but currently only resultant thickness as a function of time is extracted. To visualize and quantify the mechanism of de-swelling, we present a method exploiting the nanometer sensitivity of the Fourier phase in OCT data to image deformation velocities. The technique was demonstrated by non-invasively showing during de-swelling that osmotic flow through an intact epithelium is negligible and removing the endothelium approximately doubled the initial flow at that interface. The increased functional data further enabled the validation of a mathematical model of the cornea. Included is an efficient method of measuring high temporal resolution (1 minute demonstrated) corneal thickness, using automated collection and semi-automated graph search segmentation. These methods expand OCT capabilities to measure volume change processes for tissues and materials.

## 1. Introduction

In the human eye, the majority of light refraction is performed by the cornea. For this reason the cornea must remain transparent with regular optics and to achieve this it has a homeostatic mechanism to control hydration and thickness [1]. Several diseases and dystrophies affect the cornea leading to loss of visual acuity or blindness. Scarring of the cornea causing opacity has been identified by the World Health Organisation as the fourth leading cause of blindness worldwide, with an estimated 1.5 to 2.0 million new cases every year [2]. In the majority of cases, the only currently available curative treatment is to transplant corneal tissue from a deceased donor. In the single year of 2012, it was calculated that at least 184,576 corneal transplants were carried out worldwide [3]. The discrepancy between corneal blindness and corneal transplantation arises because access to this sight-saving surgery is limited, even in developed countries, by a major shortfall of donor tissue, with only 1 suitable cornea supplied for every 70 needed worldwide [3]. Furthermore, corneal transplant failure is a significant problem, with an overall 5 year actuarial survival of 90% [4], and virtually all will fail within 30 years [5]. To ensure these discordant rates do not rise further, not only does donation need to match the shortfall, but there also needs to be efficient and reliable quality screening of the donor tissue. Methods to measure corneal hydration and its mechanism have two separable application areas, firstly to assess the quality of donor tissue and secondly in the study and assessment of corneal diseases such as Fuchs dystrophy [6, 7].

The storage [8] and preparation [9–15] of donor human corneal tissue are important processes in achieving the best optical results and reducing rates of failure after corneal transplantation. A common method of donor cornea preservation is by storage at normothermic temperature in tissue culture [16]. This technique permits preservation of the vital corneal endothelial layer for up to one month [17]. Immersion in culture media during storage inhibits the mechanisms that maintain corneal hydration and this leads to swelling of the donor cornea to approximately double its *in vivo* thickness [18]. Transplantation of a swollen cornea causes a misalignment between one or both surfaces of the transplant compared with the recipient tissue. To counteract this, donor corneas are “de-swelled” before use in a hypertonic solution, usually containing dextran, which removes the excess water from the cornea by osmosis. The health of the endothelial layer affects the de-swelling rate [19] and conversely, the de-swelling process can damage the health of the donor tissue [20]. For these reasons there has been substantial research to optimise the de-swelling time [9, 21], improve the method [10], or use alternative osmotic agents such as poloxamers or PEG [11, 12]. As well as moving this research forward, a new method to measure and image the de-swelling process could be used to screen potential donor tissue by identifying abnormalities in behaviour.

Optical coherence tomography (OCT) is a technique that can be used safely and non-invasively to inspect corneas inside eye bank storage vials with no manipulation and no risk of contamination [22]. There has been limited use of OCT in eye banking [9, 23–25]. Conventional optical microscopy is customarily used to review the health of the endothelial layer, and OCT [26], or ultrasonic pachymetry [27], used to measure corneal thickness. Although OCT has been increasingly used for studies of corneal swelling and de-swelling [9, 11, 13, 24, 25, 28–30], the method gave thickness at the time of measurement, but has not been able to provide any direct information on the physiological processes driving these changes. The processes that happen within the cornea during the de-swelling process therefore, have had to be interpreted by mathematical modelling and only corneal thickness as a function of time has been available to validate these models [31].

As well as micrometre resolution for structural tomography, the phase information within the interferogram data collected by OCT has nanometre displacement sensitivity that has previously been applied in functional imaging techniques such as profilometry/vibrometry [32], angiography/Doppler velocity measurements [33] and optical coherence elastography (OCE) [34, 35]. In Fourier domain OCT, the Fourier phase (the complex phase angle after Fourier transform) of each pixel is sensitive to relative displacements of the object(s). This ability has been used in dynamic OCE to map in cross-section the elastic properties of a cornea [36].

When any object shrinks or expands, every single point within it has a measurable velocity relative to every other point. Compared to the velocities in Doppler blood flow measurement and dynamic OCE, the relative velocities between different points in a de-swelling cornea are orders of magnitude smaller. This means that to achieve the required motion sensitivity the ideal time between consecutive A-scans is orders of magnitude longer. During this time there can be significant bulk motion of the cornea because, as in common clinical practice, rather than being physically fixed in position, it is suspended by a suture at a single point, allowing some movement within the de-swelling solution. We mimicked this arrangement in our experiments. Here we present a new method that we term, ‘*Deformation Velocity Imaging* (DVI)’. The term is derived as the technique tomograpically *images* the relative axial *velocities* between points of a (semi)-solid object caused by its inelastic *deformation*. This method uses the temporal change in the phase difference between pixels and a reference pixel, set for each A-Scan using image segmentation to be at a given depth within the object, returning the small relative axial velocities resulting from the deformation of the object but removing any bulk motion velocity, similar to methods used for Doppler OCT and OCT angiography. It is shown that these relative velocities give a qualitative picture of the de-swelling process, allow quantitative comparisons between samples and can be used to test mathematical models of the cornea.

The DVI method presented here could be implemented with a conventional scanning point OCT system. To efficiently capture consecutive A-scans at a low repetition rate, such a system could be expected to measure repeated B-scans at the same position with negligible mechanical A-Scan lateral positioning differences. However, we instead used a line field spectral domain OCT (LF-OCT) [37] which measures B-Scans without any mechanical moving parts. Such a system has previously been used for static intraocular pressure OCE measurements [38].

In addition to the new DVI method, we used an automated segmentation method for corneal thickness change. Previous corneal de-swelling studies have adopted a manual approach to measure thickness which led to low temporal sampling; the shortest period for a corneal thickness measured within a vial was 30 minutes [30]. For these thickness measurements, as well as the automated DVI processing, the anterior and posterior interface locations are required. To achieve this, image segmentation techniques [39] can rapidly extract the location of interfaces in large numbers of images, making high temporal sampling more practical and removing user subjectivity. Graph search is a graph cut method [40] that is particularly suited for the cornea [41], due to the functional nature of the solution, low computation energy, reduced optimisation and no training requirements. Combining these techniques allowed smooth, unbiased, measurement of corneal thickness change.

## 2. Methods

### 2.1 Preparation and presentation of corneas

Ethical approval (RETH000833) from the research ethics committee of the University of Liverpool was obtained for the use of human tissue. Human corneas were dissected using a clinical eye bank technique to prepare the cornea with a 1 to 3 mm scleral rim.

The three corneas used for *Sections 3.1, 3.2 and 3.4* were supplied by the NHSBT Manchester Eye Bank, UK. They were stored under normothermic tissue culture conditions for an extended period and then held for a further 23 to 25 days at ambient temperature, such that the total storage time was 49 to 98 days. As expected, histological examination after the imaging indicated that these corneas did not have an intact endothelial layer, but did retain an epithelium, albeit abnormal. Although these corneas did not have an endothelium suitable for transplantation, they were adequate to demonstrate the technique and serve as a model of stromal thickness change. The de-swelling solution used for these three corneas was 20% dextran (Mw = 450,000 to 650,000) (Sigma-Aldrich) in distilled water.

The pair of corneas used in *Section 3.3* were supplied by The Liverpool Research Eye Bank / Royal Liverpool University Hospital, UK and imaged within the routine clinical limit of 30 days (16 and 17 days in normothermic tissue culture media). High resolution OCT and histology imaging showed neither cornea retained an epithelial layer. To compare the influence of the endothelium, this was removed along with Descemet’s membrane from one cornea the day before imaging, whereas the paired cornea was left intact (again validated with higher resolution OCT and histological imaging). These corneas were then thinned using a commercial thinning media (THIN-C, Alchimia, Padova, Italy).

For the experiments, all the corneas were suspended in media by a suture at the periphery and kept located by another suture diagonally opposite attached to a stainless steel washer weight. Imaging was carried out through the glass wall of a viewing vial. Before transferring to de-swelling solution, the corneas were imaged in the tissue culture media for at least 40 minutes to ensure equilibration (no measureable inelastic deformation). For the de-swelling experiments, first the cornea and then the storage media were removed before being replaced with de-swelling solution and the cornea re-immersed within 5 minutes. After the transfer, the LF-OCT measurements were automatically taken once every minute for the first 40 to 60 minutes, and subsequently every ten minutes, up to the extent of the experiment ranging from 17 to 42 hours.

### 2.2 Line-field spectral-domain OCT

We have previously described the LF-OCT system [42]. Here we used the iVac CCD camera (frame rate 2 Hz), and 75 mm objective and 100 mm collection achromatic lenses. This system utilises a commercial Czerny-Turner spectrograph, which allowed the axial resolution and range to be changed by switching the grating on the mechanical turret. For the automatic monitoring of the de-swelling corneas it operated at low-resolution mode using a 1200 l/mm grating. The central wavelength used was 800 nm, giving an axial resolution of 12 μm in air (n_G_ = 1), 9 μm in cornea (n_G_ = 1.37), with an axial range of 5.2 mm in air (n_G_ = 1), 3.8 mm in cornea (n_G_ = 1.37), where n_G_ is group refractive index. This low-resolution mode gave higher signal per pixel, increasing the signal to noise ratio, and a higher axial range, circumventing any axial motion of the swollen corneas during the automated collection. The axial resolution value of 9 μm in cornea (n_G_ = 1.37), is sufficient for the functional DVI and measurement of the full thickness of the cornea. For higher resolution inspection of the epithelium, the system was switched to the 300 l/mm grating, though axial resolution was likely to be limited by uncorrected dispersion from the vial wall and solution.

### 2.3 Graph search segmentation of cornea

To automatically identify anterior and posterior interfaces of the cornea, we used an iterative graph search algorithm. For each cornea, the iterative process was tailored to suit the image features. Future standardised mounting of the corneas in de-swelling vials, would overcome this need to tailor the algorithm by keeping image features, such as interface reflections, at the same position within the image and allow the same algorithm parameters to be used for all measurements.

The key process that was iterated in these algorithms was graph search segmentation. This itself is a multistage process. First, the image was smoothed with a median filter. Secondly, one of two different energy functions was selected; either an inverted amplitude of the image, or the inverted magnitude of the vertical gradient. Thirdly, the chosen energy function was then smoothed again, using boxcar averaging. Fourthly, the left-right paths of minimum energy were computed using the algorithm presented by Williams et. al. 2015 [41]. To measure low energy paths across the image (e.g. anterior and posterior corneal interfaces), the minimum energy algorithm was iterated with the pixels within a set vertical range of the prior paths being blocked. The size of the median filter, boxcar and blocked vertical range were set to the lowest robust values by trial and error for each application of graph search.

The following process was used for each cornea: The appropriate vertical range to be considered was selected, then the inverted amplitude graph search was used to find the strong signals of the container interfaces and remove them from the searched image. For the three corneas with strong scattering from residual epithelium layers, the inverted amplitude search also returned the position of this layer. For the two corneas without an epithelium, the vertical gradient energy function was used to recover the profile of this interface instead. Once the approximate epithelial interface was established, this was used to aid the final segmentation by reshaping the image to this profile to make the cornea flat and discard irrelevant areas. A final run of the graph search algorithm, using the vertical gradient energy function, was then used to find the top and bottom interfaces of the cornea in the manipulated image. These interfaces were then displayed back on to the original images. The quality of the sectioning was then manually reviewed and clearly false paths manually blocked to ensure reliable sectioning.

### 2.4 Deformation velocity imaging (DVI)

In FD OCT, the product of the discrete Fourier transform is complex. The signal for each pixel, *I’_P_*, and its complex phase angle (Fourier phase), *ϕ_P_*, are given by

$$I_{P}^{΄}=A_{P}(a_{P}+b_{P}i)=A_{P}a_{P}+A_{P}b_{P}i.$$ (1)

$$\phi_{P}=\tan^{-1}\left(\frac{b_{P}}{a_{P}}\right)=\tan^{-1}\left(\frac{A_{P}b_{P}}{A_{P}a_{P}}\right).$$ (2)

Where, *A_P_*, is the magnitude of the signal giving the classical OCT images, and a_P_ and b_P_ are constants where inside the cornea, the signal in each pixel, *P*, will come from multiple scattering points, but these move approximately in unison. Hence the resultant phase behaves as if there was only a single signal source. Axial velocity of motion can be measured from the differences in the Fourier phase of a signal between consecutive measurements, *Δϕ*, by [33]

$$v=\frac{\Delta \phi \lambda }{4\pi n\Delta \tau }=\frac{d\phi }{d\tau }\frac{\lambda }{4\pi n},$$ (3)

where *λ* is the central wavelength, *n* is the medium refractive index, and *Δτ* is time difference between the two measurements. It is generalised further by considering the measurement of an average unwrapped temporal phase gradient, *dϕ/dτ*, over an arbitrary number of measurements rather than just two. The relative axial velocity between objects in each pixel, *P*, and a reference pixel, *RP*, can be calculated by

$$v_{r}=\frac{d(\phi_{P}-\phi_{RP})}{d\tau }\frac{\lambda }{4\pi n}.$$ (4)

The temporal gradient of phase change, $\frac{d(\phi_{P}-\phi_{RP})}{d\tau }$, can be calculated by linear regression of unwrapped *ϕ_P_*-*ϕ_RP_* verses *τ*, over any number of measurements. The reference pixel can be set for each A-Scan to be at a given depth within a sample, using its boundaries found by image segmentation.

In this work, the relative phase change rate was measured from the ten frames of unprocessed complex B-Scans by the following method: Firstly, to remove any bulk motion for each A-scan and make the measured axial velocity relative to a specific point, the pixel with the strongest signal within a ten pixel range of a set point in the cornea was found. For the three corneas with an intact epithelium the set point was the epithelial interface and for the two corneas without epithelium, to prevent aliasing of the phase signal at either interface, the equidistant position between anterior and posterior surfaces was used instead. The phase value of the found pixel was then subtracted from the phase value of all the pixels in that A-scan. Secondly, to recover the change from the first frame, for each pixel the phase value from the first frame was then subtracted for all frames. Thirdly, for each pixel the phase change was unwrapped over the ten frames. Fourthly, for each pixel the temporal gradient of the phase change over the ten frames was taken ($\frac{d(\phi_{P}-\phi_{RP})}{d\tau }$). For the images, only the pixels in the segmented cornea were taken and then the image smoothed to reduce noise. This smoothing was not carried out prior to axial averaging.

The minimum detectable relative axial deformation velocity is determined by its noise. Estimated spatially over an area of an equilibrated swollen cornea in tissue culture, the standard deviation of the raw pixel values was 12 nm/s with correction for assumed corneal refractive index of n = 1.37, spatially smoothed images (boxcar type averaging) was 3 nm/s (n = 1.37) and axial averaged data was 0.7 nm/s (n = 1.37). The self-evident approaches to improving these values further are increasing the OCT SNR and increasing the measurement time, so that more deformation displacement occurs. The maximum measureable deformation velocity was capped by the Nyquist (aliasing) limit, of the 2 Hz frame rate, to 290 nm/s (n = 1.37). Two potential strategies exist for increasing this further, firstly the standard one of increasing measurement rate. Secondly, as the OCT image signal from the cornea (and any other mildly scattering sampling) is continuous, the aliasing of distant axial points may be overcome by introducing spatial unwrapping from the reference pixel.

### 2.5 De-swelling mathematical model

Previously, only the resultant thickness data has been used to validate swelling models of the cornea [31, 43]. To mathematically model the first stage of the de-swelling process, for the first three corneas, we took the model given by Li et al. [31]. As we limited the modelling to just the rapid initial osmotic pressure driven de-swelling flow, we ignored the effect of solute movement throughout. The model has stroma medium permeability, K, as a function of the ratio of wet to dry mass (hydration), $\frac{M_{W}}{M_{D}}$, however, to reduce calculation complexity for this proof of principle demonstration, we simplified it to a single constant value. We used model elements of equal dry thickness and a density of 1g/cm^3^ for dry and water components. CGS units were used to match the previous work. The differential model was resolved numerically in MATLAB R2015aSP1 (MathWorks, Natick, MA). The values of the constants were recovered by manually fitting the model to give the best fit to the mean axial deformation velocity measurement at three times.

## 3. Results and discussions

### 3.1 Semi-automated de-swelling corneal thickness measurement by graph search

Figure 1(a)-1(d)

**Fig. 1:**
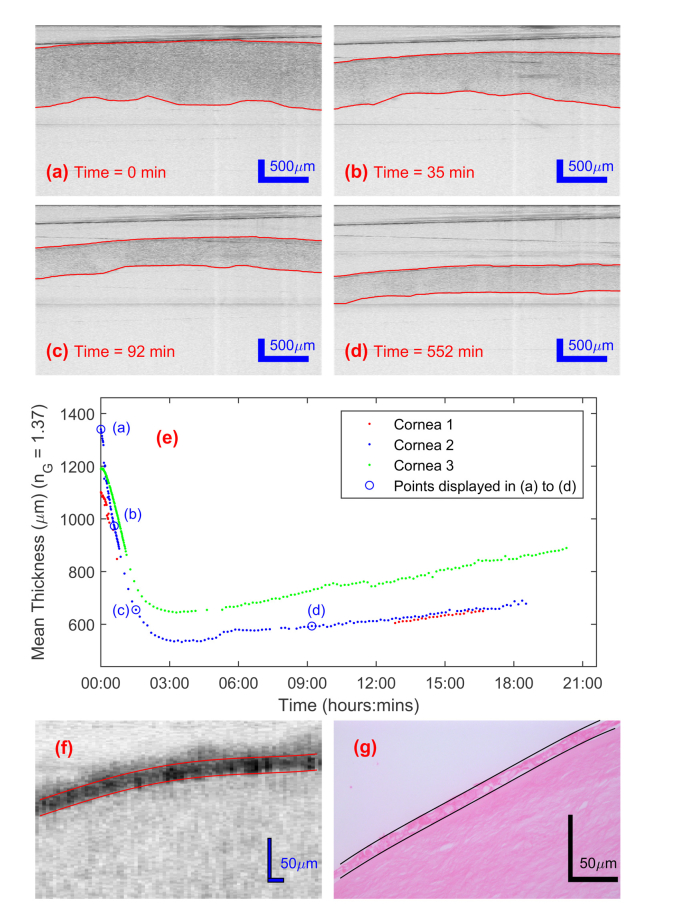
(a-d) OCT images and graph search segmentation (red lines) of a de-swelling human cornea within a vial at four different times. Visualization 1 shows a video stream of all 156 successive images taken during a de-swelling experiment of 18.5 hours. (e) Measured mean thickness of the de-swelling of three corneas. The locations of the four images in (a-d) are marked with blue circles. A constant group refractive index of 1.37 has been assumed, though the changing composition of the cornea would lead to small variances in this value. (f-g) Representative higher resolution OCT (f) and haematoxylin and eosin histological images (g) zoomed to show the residual epithelial layer (highlighted with red and black lines respectively).

are four examples (from Visualization 1) of the automatically collected B-scan structural images, and their automated graph search segmentation of the anterior and posterior interface, from one de-swelling experiment. Figure 1(e) shows the mean thickness measured by the automated segmentation for three corneas over 18 hours of transfer to a de-swelling solution. All three corneas followed the same thickness profile, with an initially rapid decrease for approximately 3 hours caused by the osmotic de-swelling due to the hypertonic dextran solution. After this minimum, there was a slower increase in the thickness of the cornea [19], which was associated with dextran diffusing into the cornea [44], changing the osmotic balance. The temporal sampling period of the thickness measurements of de-swelling corneas, by this automated method, is much smaller (1 versus 30 minutes [30]) than has previously been presented. In addition, the automated measurement reduced lateral misalignments between consecutive images and the automated segmentation removed subjectivity, leading to low noise in the thickness values. As a result, the initial rapid decrease in thickness was accurately recorded without the need for interpolation. As will be discussed below, this detailed temporal thickness data can be compared directly with the trend predicted by mathematical models of the cornea. One minute sampling was chosen as sufficient to follow the initial rapid de-swelling, although the automated collection and segmentation would allow faster sampling with minimal operator intervention; the ultimate sampling rate is limited by camera speed and available data storage. The automated data collection for cornea 1 (red dots) is incomplete due to a computer failure that did not reoccur. However, for the time periods successfully collected, the data closely matches its pair, cornea 2 (blue dots). OCT structural imaging can also resolve the separate layers of the cornea. For example, Fig. 1(f) is a high resolution OCT image of the anterior surface of the cornea showing a scattering residual epithelial layer. The epithelial layer was then confirmed by histological staining (Fig. 1(g)) after the completion of the experiment.

### 3.2 Deformation velocity imaging (DVI)

**Fig. 2:**
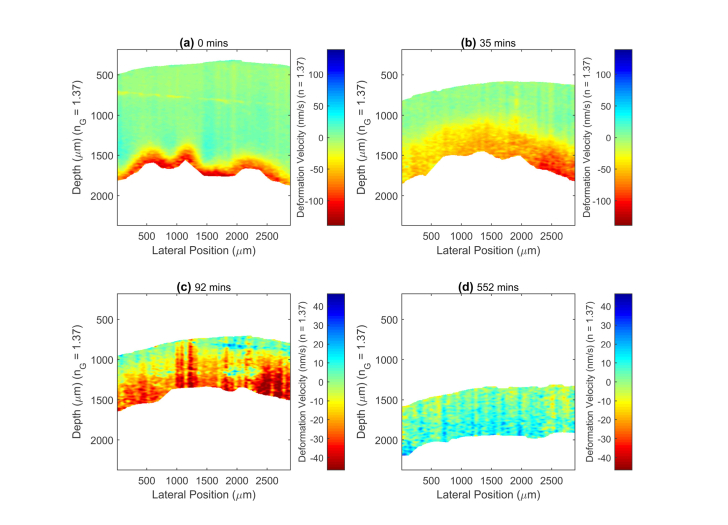
The Deformation Velocity Images (DVI) at four times during the de-swelling of a cornea with an intact epithelial layer. Visualization 2 shows the sequence of images up to 552 minutes.

The Fourier phase information from the 10 images automatically collected over 5 seconds at each sampling time was used to produce the deformation velocity images. The local strain rate, which is the fractional rate of expansion or shrinkage, is proportional to the axial gradient in these images (axial velocity gradient). Given approximate conservation of volume (*i.e.* Starting with 1.5 cl of swollen cornea, then removing 0.5 cl of fluid will leave 1 cl of cornea remaining. Any non-ideal solution behaviour would lead to some negligible deviation from this.) and taking the de-swelling as a one-dimensional process, the image amplitude is then linearly related to the osmotic flow rate. Note it is not a direct measure of the flow of the transparent fluid, but rather of the resulting reflex motion of the semi-solid matrix of the cornea. Figure 2

and Visualization 2 shows the corresponding DVI versions of the structural images given in Fig. 1(a)-1(d) and Visualization 1 respectively. At the start of the experiment (Fig. 2(a)) the image shows a large deformation velocity (flow rate) and strong axial velocity gradient (strain i.e. shrinkage) at the endothelial interface of the cornea, while no deformation velocity or axial velocity gradient is visible above noise at other depths. This initiation of shrinkage at this interface means that the largest osmotic flow must have occurred via this interface. The DVI images of these three corneas showed no indication of significant de-swelling initiating from the epithelial interface which indicates the low hydraulic conductivity of this layer [31]. Higher resolution OCT structural imaging and histology after the imaging (Fig. 1(f)-1(g)) confirmed the presence of a residual epithelial layer.

By 35 minutes (Fig. 2(b)) the front of the de-swelling moved further into the cornea, which was shown by a larger proportion of the cornea having an axial velocity gradient in the deformation velocity (strain i.e. shrinkage due to flow gradient). However, the image amplitude at the endothelial interface is slightly reduced, indicating that the total net flow out of the cornea has decreased. By 92 minutes (Fig. 2(c)) the amplitude of deformation velocities, thus net flow rate and overall shrinkage, had significantly decreased and the average axial velocity gradient of the deformation velocity image (strain rate) was more uniform throughout the depth of the cornea. At 550 minutes (Fig. 2(d)) the axial deformation velocities and their axial velocity gradient is reversed, indicating corneal re-swelling as shown in the thickness measurements (Fig. 1(e)). With the distinctive traits identified in the DVI images, the stage of de-swelling of any cornea could be identified quasi-instantaneously by the technique. In addition, as the DVI information is quantitative it could be used to measure the rate of re-swelling, which has been linked to the health of the vital endothelial layer [19]. Currently, to supply corneal tissue for transplantation, the health of the endothelium is only assessed by its visual appearance or cellular level staining [45]. The experimental results reported here suggest that DVI has the potential to directly measure the functional performance of the endothelium.

For this phase method, to ensure significant phase change while not encountering aliasing, the measurement rate of 2 Hz was appropriate for the magnitude of the deformation motion of de-swelling. The ten consecutive images used for each measurement, meant a total measurement time of 5 seconds for each deformation velocity image. This is small compared to the time frame of swelling or de-swelling process, so can be regarded as quasi-instantaneous.

It has previously been demonstrated that OCT can also be used to perform the count and shape assessment of endothelial cells [46–48]. With the development of OCT, a single system carrying out both *en face* endothelial cell counts and DVI measurements is a possibility. This would be a powerful tool enabling an eye bank to rapidly perform a rigorous analysis of both corneal endothelial appearance and function, and hence donor tissue transplant suitability.

Within some of the DVI images artefacts can be seen due to the overlay of folded interface signals on to the OCT image data, such as the slanted horizontal line at ~750 μm depth in Fig. 2(a). The overlay of such signals should be avoidable with further experimental refinement.

Within this section and 3.3 we have shown that imaging the relative axial velocities due to the inelastic deformation of a cornea during de-swelling gives a qualitative tomographic picture of the process that was not previously possible. In section 3.3, we show the axial averaged values can compare between samples quantitatively and in 3.4 the quantitative values can be compared directly to mathematical models. Conceptually the strain rate (shrinkage) is also important and could be imaged by calculation of axial gradient of velocity. However, taking the differential of the data would be expected to adversely affect the signal to noise ratio and/or resolution. In OCE, where the imaging of strain [49] is essential, improved methods have been developed to enhance performance. The strategies include: measuring local spatial phase gradient to return strain instead of displacement [49]; replacing the spatial averaging of the end result with weighted averaging [49] or earlier complex data averaging [50, 51] to decrease noise; physically adopting a common path reference surface in the interferometer [50] to minimise path length noise; and combining the phase method with speckle tracking [52] to increase dynamic range. Some of these strategies applied to DVI may improve imaging performance further and/or give direct images of strain rate instead.

As well as for the cornea, the technique may also be of value in the measurement of swelling processes in other tissues [53] or bio-materials [54], for the study of transport mechanisms or the differentiation of tissue. Outside of medicine, the technique could be applied to the measurement of thermal expansion processes in materials [55] or measuring how dry coating layers are by detecting the rate at which they change thickness [56].

Like standard phase based Doppler OCT and OCE methods, the described DVI method is only sensitive to changes in one dimension (axial). In Doppler OCT the compositing of simultaneous measurements with different optical axes [57] is being developed to measure three-dimensional change and such a method may be applied to DVI.

### 3.3 The effect of endothelial or epithelial layers

The barrier properties of the epithelial and endothelial layers are crucial to the de-swelling process of donor corneas. To examine the effect of their presence, a pair of donor human corneas both without epithelium and one without endothelium and Descemet’s membrane were de-swelled in a commercial de-swelling medium (THIN-C).

**Fig. 3:**
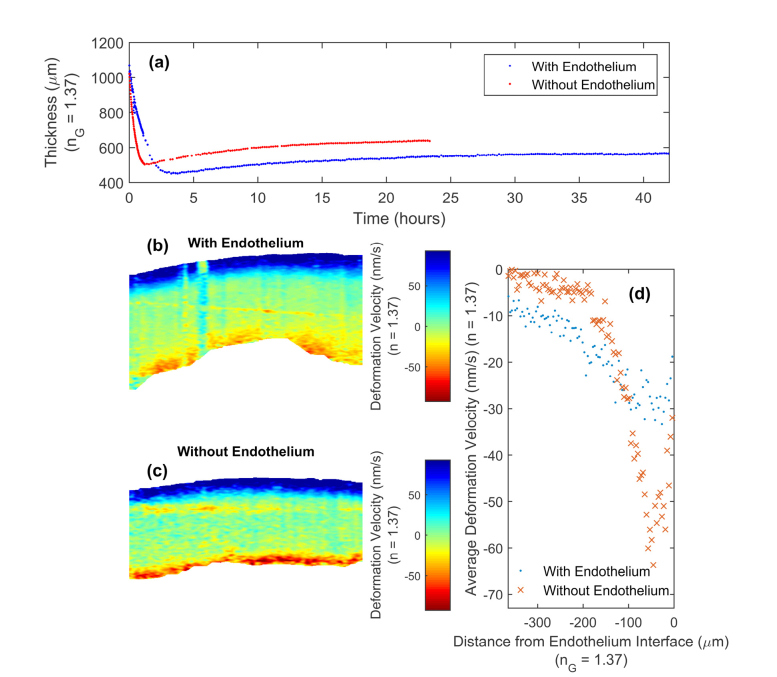
(a) Thickness results for a pair of corneas, one with and one without endothelial and Descemet’s layers. (b,c) Comparison DVI images at 0 minutes for both corneas. (d) Averaged, from the endothelial interface, deformation velocities for both corneas.

To study the effect of the endothelial layers, Fig. 3(a)

shows the thickness measurements for both corneas. As expected, the removal of the endothelial layer from the cornea (i.e. one of the barriers to flow) led to a more rapid initial thinning. Furthermore, this cornea also began to re-swell sooner, commencing at a greater minimum thickness, and rising to a larger final thickness compared to the cornea with an intact endothelial layer. This is likely due to the endothelial and Descemet’s layers also being a barrier to dextran diffusing into the stroma. The removal of this barrier means that the dextran can diffuse more easily into the corneal stroma, equalising the osmotic pressure more rapidly. These measured thickness curves are visually similar to those produced by Schnitzler et al. [19], where damaged and undamaged endothelial layers were also compared. The high temporal sampling produced by our automatic collection and image segmentation meant that the detailed temporal thickness relationship was measured directly without relying on linear regression interpolation.

In order to visualise which corneal interfaces are involved and the relative magnitudes of de-swelling, Fig. 3(b), 3(c) shows the initial DVI images for the two corneas. For the endothelial interface, the cornea with an intact endothelial layer had a lower average magnitude of deformation velocity than the one without the endothelium. This corresponds with a greater axial velocity gradient in the image (strain rate), which is caused by a larger osmotic flow rate due to the removal of the endothelial barrier. In order to have a quantitative comparison, the mean deformation velocities at the endothelial interface for the two corneas were calculated for each distance from the endothelial interface. As shown in Fig. 3(d), the cornea without the endothelial layer had approximately twice the maximum deformation velocity magnitude at this interface.

Comparison of DVI results of corneas with an epithelial layer (Fig. 2(a)) and those without epithelial layer (Figs. 3(b), 3(c)) showed a difference at the epithelial interface, demonstrating the effect of the epithelial layer. With these two corneas the de-swelling process occurred at the epithelial, as well as the endothelial, interface. Moreover, the measured deformation velocity difference, for these two corneas, appeared larger at the epithelial side even with the endothelium and Descemet’s membrane removed. Even if both epithelial and endothelial surfaces pose no effective barrier to water and dextran, their osmotic flow rates may still differ because of differences in the biomechanics between anterior and posterior stroma [58].

### 3.4 Use of DVI to validate cornea mathematical models

To understand how a cornea functions, particularly the de-swelling behaviour, a mathematical model of fluid flow has to be derived based on fluid dynamics. In this section we demonstrate that DVI data can be used to give a more direct experimental validation and testing of these theoretical models. The DVI data from *Section 3.2* was further used to validate the mechanism underlying a mathematical model of the de-swelling process. The parameter values used were fitted empirically using the DVI data at three time points and then compared to reference values. Table 1

**Table 1:** Parameter values, and comparison referenced values, used in mathematical model.

Parameter	Fitted Values	Reference Values
Fatt and Goldstick 1965 [59] empirical constant, *C* (dyne/cm^2^).	N/A ^A^	2.41 x 10^6^ [59].
Flow conductivity, $\frac{k}{\mu }$ (cm^4^/(s.dyne)).	10 x 10^−12^.	0.3 to 29 x 10^−12^ [31].
Initial hydration, $\frac{M_{W}}{M_{D}}$.	7.6	1.5 to 8 [43]. ^B^
De-swollen hydration, $\frac{M_{W}}{M_{D}}$.	2.44	As above.
Endothelium membrane hydraulic conductivity, *L* (cm^3^/(s.dyne)).	179 x 10^−12^	15.8 x 10^−12^ [31].

^A^ Not mathematical separable from model parameters, taken as equal to reference.

^B^ Plausible hydration range (rabbit model).

**Fig. 4:**
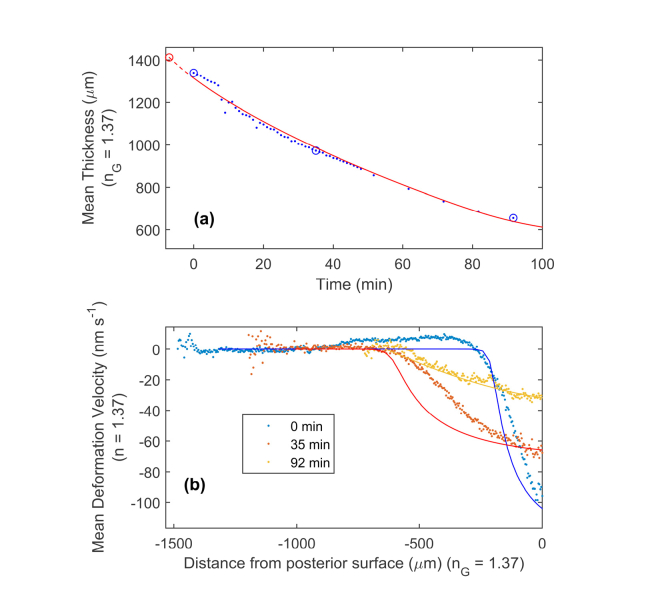
(a) Comparison of measured (blue points) and modelled (red line) mean thicknesses of a cornea over the first 92 minutes of a de-swelling experiment. The offset starting time and thickness of the model is shown by the red circle, with the red dashed line showing the modelled thickness relationship before the first measurement. The blue circles mark the three measurements shown in (b). (b) Comparison of the measured (points) and modelled (lines) mean deformation velocities as a function of distance from the endothelium interface, at three chosen times. The parameters for the model were fitted using these three measured deformation velocity data sets. The fitted parameters are given in Table 1.

gives the referenced and fitted values for the key parameters. Apart from the endothelial membrane hydraulic conductivity, the fitted values are within the expected referenced range. The fitted endothelial layer hydraulic conductivity is an order of magnitude higher than predicted for a normal cornea. This is likely due to the poor endothelial integrity of this cornea, as revealed histologically. Though OCT and histology results showed the presence of a residual epithelial layer, endothelial cells were only seen in a small fraction of the histological images and, where they were present, they were only individual cells rather than a complete layer. This degradation of the endothelial layer is the likely cause of the order of magnitude increase in porosity of the layer. Figure 4

(top) shows the automatically measured thickness results over the first 92 minutes for one of the corneas and the predicted thickness from the mathematical model with fitted constant values. Although there are small systematic discrepancies, the model does give an accurate prediction of thickness, which is encouraging as the model parameters were only fitted using the mean thickness and DVI results at three time points. From these three time points, the model predicted the thickness and the state of internal processes at any point in between. Figure 4 (bottom) shows the measured depth (from endothelial interface) average and the modelled DVI results. Comparison between the model and data show macroscopic agreement, substantiating the working principles of the model. Issues with this simple model, however, are clear, with the more abrupt changes with the model. Refinement of the model, such as expansion from 1D to 3D with non-uniform thickness and re-inclusion of features such as solute effects (particularly dextran diffusion) and dependence of *k* on$\frac{M_{W}}{M_{D}}$, may lead to closer matching of function and its application over longer time periods. The increased amount of information that DVI provides may in future allow improved models of the cornea to be developed, increasing the understanding of the de-swelling process and corneal physiology.

## 4. Conclusion

We have presented a new method, DVI, that quasi-instantaneously maps in cross-section the deformation velocities of corneal tissue undergoing volume change and in doing so have also improved the temporal sampling by using automated collection and segmentation. The DVI results showed at which interfaces and at what magnitude de-swelling (osmotic flow) occurs and represents a direct non-invasive measure of the local flow mechanisms. This data allowed the validation of a current mathematical model of the cornea and may aid the development of improved models.

The results demonstrate that DVI not only has immediate application for corneal swelling and de-swelling research, but may have a potential application in the quality control of donor corneas for transplantation, by rapidly assessing the functional barrier performance of the epithelial or endothelial layer. We also see potential application of the technique to other tissues, biomedical materials, and, outside of medicine, thermal and coating drying, where non-invasive monitoring of inelastic deformation is of value.

## References

[1] Friedman M. H., “A Quantitative Description of Equilibrium and Homeostatic Thickness Regulation in the In Vivo Cornea. I. Normal Cornea,” Biophys. J. 12(6), 648–665 (1972). 5063840 10.1016/S0006-3495(72)86110-1 PMC1484153

[2] World Health Organisation, “Prevention of Blindness and Visual Impairment Programme: Priority eye diseases; Corneal opacities,” (Retrieved from http://www.who.int/blindness/causes/priority/en/index8.html, 19/09/2017).

[3] Gain P. Jullienne R. He Z. Aldossary M. Acquart S. Cognasse F. Thuret G., “Global Survey of Corneal Transplantation and Eye Banking,” JAMA Ophthalmol. 134(2), 167–173 (2016). 26633035 10.1001/jamaophthalmol.2015.4776

[4] Larkin D. F. Mumford L. L. Jones M. N. NHS Blood and Transplant Ocular Tissue Advisory Group and Contributing Ophthalmologists (OTAG Audit Study 7), “Centre-specific variation in corneal transplant outcomes in the United Kingdom,” Transplantation 91(3), 354–359 (2011). 21079549 10.1097/TP.0b013e318201ac62

[5] Williams K. A. Keane M. C. Galettis R. A. Jones V. J. Mills R. A. Coster D. J., “The Australian Corneal Graft Registry 2015 Report,” (2015).

[6] Wacker K. McLaren J. W. Kane K. M. Baratz K. H. Patel S. V., “Corneal Hydration Control in Fuchs’ Endothelial Corneal Dystrophy,” Invest. Ophthalmol. Vis. Sci. 57(11), 5060–5065 (2016). 27661858 10.1167/iovs.16-20205 PMC5040190

[7] Nielsen E. Ivarsen A. Erlandsen M. Hjortdal J., “Evaluation of Endothelial Pump Function in Fuchs Endothelial Dystrophy Before and After Endothelial Keratoplasty,” Cornea 35(6), 878–883 (2016). 27055221 10.1097/ICO.0000000000000821

[8] Jeng B. H., “Preserving the cornea: corneal storage media,” Curr. Opin. Ophthalmol. 17(4), 332–337 (2006). 16900023 10.1097/01.icu.0000233950.63853.88

[9] Wolf A. H. Welge-Lüssen U. C. Priglinger S. Kook D. Grueterich M. Hartmann K. Kampik A. Neubauer A. S., “Optimizing the Deswelling Process of Organ-Cultured Corneas,” Cornea 28(5), 524–529 (2009). 19421045 10.1097/ICO.0b013e3181901dde

[10] Bucher F. Roters S. Mellein A. Hos D. Heindl L. M. Cursiefen C. Hermann M., ““OSMO-UT-DSAEK” using THIN-C medium,” Graefes Arch. Clin. Exp. Ophthalmol. 251(9), 2181–2185 (2013). 23907483 10.1007/s00417-013-2434-0

[11] Lie J. T. Monnereau C. Groeneveld-van Beek E. A. van der Wees J. Frank J. Bruinsma M. Melles G. R. J., “Dehydration of corneal anterior donor tissue with polyethylene glycol (PEG)-enriched media,” Cell Tissue Bank. 16(3), 399–409 (2015). 25432155 10.1007/s10561-014-9484-3

[12] Zhao M. Thuret G. Piselli S. Pipparelli A. Acquart S. Peoc’h M. Dumollard J. M. Gain P., “Use of poloxamers for deswelling of organ-cultured corneas,” Invest. Ophthalmol. Vis. Sci. 49(2), 550–559 (2008). 18234998 10.1167/iovs.07-1037

[13] Rossi M. Mistò R. Gatto C. Garimoldi P. Campanelli M. D’Amato Tóthová J., “Protective Effects of Deswelling on Stromal Collagen Denaturation After a Corneal Femtosecond Laser Cut,” Invest. Ophthalmol. Vis. Sci. 54(6), 4148–4157 (2013). 23702778 10.1167/iovs.12-10818

[14] Romano V. Steger B. Chen J. Y. Hassaan S. Batterbury M. Willoughby C. E. Ahmad S. Elsheikh A. Kaye S. B., “Reliability of the Effect of Artificial Anterior Chamber Pressure and Corneal Drying on Corneal Graft Thickness,” Cornea 34(8), 866–869 (2015). 25933404 10.1097/ICO.0000000000000451

[15] Romano V. Steger B. Myneni J. Batterbury M. Willoughby C. E. Kaye S. B., “Preparation of ultrathin grafts for Descemet-stripping endothelial keratoplasty with a single microkeratome pass,” J. Cataract Refract. Surg. 43(1), 12–15 (2017). 28317665 10.1016/j.jcrs.2016.12.009

[16] Pels E. Rijneveld W. J., “Organ culture preservation for corneal tissue. Technical and quality aspects,” Dev. Ophthalmol. 43, 31–46 (2009). 19494635 10.1159/000223837

[17] Armitage W. J. Dick A. D. Bourne W. M., “Predicting endothelial cell loss and long-term corneal graft survival,” Invest. Ophthalmol. Vis. Sci. 44(8), 3326–3331 (2003). 12882777 10.1167/iovs.02-1255

[18] Nilius R. Redbrake C. Salla S. Reim M., “Influence of preparation and storage on human corneal thickness,” Vision Res. 35, 173 (1995).

[19] Schnitzler A. C. Salla S. Hamsley N. Flammersfeld A. Fuest M. Walter P. Hermel M., “Role of the endothelial layer in the deswelling process of organ-cultured human corneas before transplantation,” Cornea 35(9), 1216–1221 (2016). 27429079 10.1097/ICO.0000000000000937

[20] Smith V. A. Johnson T. K., “Identification and evaluation of a thinning agent compatible with MegaCell DCS, an animal product-free corneal storage medium,” Graefes Arch. Clin. Exp. Ophthalmol. 250(12), 1777–1786 (2012). 23011001 10.1007/s00417-012-2126-1 PMC3501186

[21] Pels E. Schuchard Y., “The effects of high molecular weight dextran on the preservation of human corneas,” Cornea 3(3), 219–227 (1985). 6085762

[22] Neubauer A. S. Priglinger S. G. Thiel M. J. May C. A. Welge-Lüssen U. C., “Sterile structural imaging of donor cornea by optical coherence tomography,” Cornea 21(5), 490–494 (2002). 12072724 10.1097/00003226-200207000-00010

[23] Bald M. R. Stoeger C. Galloway J. Tang M. Holiman J. Huang D., “Use of fourier-domain optical coherence tomography to evaluate anterior stromal opacities in donor corneas,” J. Ophthalmol. 2013, 397680 (2013). 23606944 10.1155/2013/397680 PMC3625538

[24] D. Nankivil, A. Pocobelli, S. Yoo, and J.-M. Parel, “Measuring human donor posterior corneal lenticules with optical coherence tomography,” SPIE Newsroom (2011), http://spie.org/newsroom/3836-measuring-human-donor-posterior-corneal-lenticules-with-optical-coherence-tomographyretrieved26/05/2017.

[25] Amato D. Lombardo M. Oddone F. Nubile M. Colabelli Gisoldi R. A. Villani C. M. Yoo S. Parel J. M. Pocobelli A., “Evaluation of a new method for the measurement of corneal thickness in eye bank posterior corneal lenticules using anterior segment optical coherence tomography,” Br. J. Ophthalmol. 95(4), 580–584 (2011). 21113073 10.1136/bjo.2010.190595

[26] Ruzza A. Parekh M. Ferrari S. Salvalaio G. Nahum Y. Bovone C. Ponzin D. Busin M., “Preloaded donor corneal lenticules in a new validated 3D printed smart storage glide for Descemet stripping automated endothelial keratoplasty,” Br. J. Ophthalmol. 99(10), 1388–1395 (2015). 25926517 10.1136/bjophthalmol-2014-306510

[27] Kelliher C. Engler C. Speck C. Ward D. Farazdaghi S. Jun A. S., “A comprehensive analysis of eye bank-prepared posterior lamellar corneal tissue for use in endothelial keratoplasty,” Cornea 28(9), 966–970 (2009). 19724219 10.1097/ICO.0b013e31819c4fcf

[28] Hutchings N. Simpson T. L. Hyun C. Moayed A. A. Hariri S. Sorbara L. Bizheva K., “Swelling of the Human Cornea Revealed by High-speed, Ultrahigh-Resolution Optical Coherence Tomography,” Invest. Ophthalmol. Vis. Sci. 51(9), 4579–4584 (2010). 20435597 10.1167/iovs.09-4676

[29] Wu Y. Clarke D. Mathew A. Nicoud I. Li X., “Noninvasive optical coherence tomography monitoring of structure and hydration changes of human corneas in different preservation media,” J. Biomed. Opt. 16(2), 026015 (2011). 21361699 10.1117/1.3541792 PMC3055587

[30] Choi C. Y. Youm D. J. Kim M. J. Tchah H., “Changes in Central Corneal Thickness of Preserved Corneas Over Time Measured Using Anterior Segment Optical Coherence Tomography,” Cornea 28(5), 536–540 (2009). 19421043 10.1097/ICO.0b013e31819140a9

[31] Li L. Y. Tighe B. J. Ruberti J. W., “Mathematical modelling of corneal swelling,” Biomech. Model. Mechanobiol. 3(2), 114–123 (2004). 15378390 10.1007/s10237-004-0054-7

[32] Yaqoob Z. Choi W. Oh S. Lue N. Park Y. Fang-Yen C. Dasari R. R. Badizadegan K. Feld M. S., “Improved phase sensitivity in spectral domain phase microscopy using line-field illumination and self phase-referencing,” Opt. Express 17(13), 10681–10687 (2009). 19550464 10.1364/oe.17.010681 PMC2844447

[33] Leitgeb R. A. Werkmeister R. M. Blatter C. Schmetterer L., “Doppler Optical Coherence Tomography,” Prog. Retin. Eye Res. 41, 26–43 (2014). 24704352 10.1016/j.preteyeres.2014.03.004 PMC4073226

[34] Schmitt J., “OCT elastography: imaging microscopic deformation and strain of tissue,” Opt. Express 3(6), 199–211 (1998). 19384362 10.1364/oe.3.000199

[35] Larin K. V. Sampson D. D., “Optical coherence elastography - OCT at work in tissue biomechanics [Invited],” Biomed. Opt. Express 8(2), 1172–1202 (2017). 28271011 10.1364/BOE.8.001172 PMC5330567

[36] Wang S. Larin K. V., “Noncontact depth-resolved micro-scale optical coherence elastography of the cornea,” Biomed. Opt. Express 5(11), 3807–3821 (2014). 25426312 10.1364/BOE.5.003807 PMC4242019

[37] Zuluaga A. F. Richards-Kortum R., “Spatially resolved spectral interferometry for determination of subsurface structure,” Opt. Lett. 24(8), 519–521 (1999). 18071558 10.1364/ol.24.000519

[38] De la Torre-Ibarra M. H. Ruiz P. D. Huntley J. M., “Double-shot depth-resolved displacement field measurement using phase-contrast spectral optical coherence tomography,” Opt. Express 14(21), 9643–9656 (2006). 19529355 10.1364/oe.14.009643

[39] Pal N. R. Pal S. K., “A review on image segmentation techniques,” Pattern Recognit. 26, 1277–1294 (1993).

[40] Boykov Y. Veksler O. Zabih R., “Fast approximate energy minimization via graph cuts,” IEEE Trans. Pattern Anal. Mach. Intell. 23, 1222–1239 (2001).

[41] Williams D. Zheng Y. Bao F. Elsheikh A., “Fast segmentation of anterior segment optical coherence tomography images using graph cut,” Eye Vis (Lond) 2, 1 (2015). 26605357 10.1186/s40662-015-0011-9 PMC4657268

[42] Lawman S. Dong Y. Williams B. M. Romano V. Kaye S. Harding S. P. Willoughby C. Shen Y. C. Zheng Y., “High resolution corneal and single pulse imaging with line field spectral domain optical coherence tomography,” Opt. Express 24(11), 12395–12405 (2016). 27410154 10.1364/OE.24.012395

[43] Hedbys B. O. Mishima S., “The thickness-hydration relationship of the cornea,” Exp. Eye Res. 5(3), 221–228 (1966). 5914654 10.1016/s0014-4835(66)80010-6

[44] Redbrake C. Salla S. Nilius R. Becker J. Reim M., “A histochemical study of the distribution of dextran 500 in human corneas during organ culture,” Curr. Eye Res. 16(5), 405–411 (1997). 9154377 10.1076/ceyr.16.5.405.7044

[45] Stocker F. W. King E. H. Lucas D. O. Georgiade N., “A comparison of two different staining methods for evaluating corneal endothelial viability,” Arch. Ophthalmol. 76(6), 833–835 (1966). 4162673 10.1001/archopht.1966.03850010835010

[46] Akiba M. Maeda N. Yumikake K. Soma T. Nishida K. Tano Y. Chan K. P., “Ultrahigh-resolution imaging of human donor cornea using full-field optical coherence tomography,” J. Biomed. Opt. 12(4), 041202 (2007). 17867791 10.1117/1.2764461

[47] Tankam P. He Z. Chu Y. J. Won J. Canavesi C. Lepine T. Hindman H. B. Topham D. J. Gain P. Thuret G. Rolland J. P., “Assessing microstructures of the cornea with Gabor-domain optical coherence microscopy: pathway for corneal physiology and diseases,” Opt. Lett. 40(6), 1113–1116 (2015). 25768195 10.1364/OL.40.001113 PMC4429128

[48] Ang M. Konstantopoulos A. Goh G. Htoon H. M. Seah X. Lwin N. C. Liu X. Chen S. Liu L. Mehta J. S., “Evaluation of a micro-optical coherence tomography for the corneal endothelium in an animal model,” Sci. Rep. 6, 29769 (2016). 27416929 10.1038/srep29769 PMC4945948

[49] Kennedy B. F. Koh S. H. McLaughlin R. A. Kennedy K. M. Munro P. R. T. Sampson D. D., “Strain estimation in phase-sensitive optical coherence elastography,” Biomed. Opt. Express 3(8), 1865–1879 (2012). 22876350 10.1364/BOE.3.001865 PMC3409705

[50] Kennedy B. F. McLaughlin R. A. Kennedy K. M. Chin L. Curatolo A. Tien A. Latham B. Saunders C. M. Sampson D. D., “Optical coherence micro-elastography: mechanical-contrast imaging of tissue microstructure,” Biomed. Opt. Express 5(7), 2113–2124 (2014). 25071952 10.1364/BOE.5.002113 PMC4102352

[51] Zaitsev V. Y. Matveyev A. L. Matveev L. A. Gelikonov G. V. Sovetsky A. A. Vitkin A., “Optimized phase gradient measurements and phase-amplitude interplay in optical coherence elastography,” J. Biomed. Opt. 21(11), 116005 (2016). 27824215 10.1117/1.JBO.21.11.116005

[52] Zaitsev V. Y. Matveyev A. L. Matveev L. A. Gelikonov G. V. Gubarkova E. V. Gladkova N. D. Vitkin A., “Hybrid method of strain estimation in optical coherence elastography using combined sub-wavelength phase measurements and supra-pixel displacement tracking,” J. Biophotonics 9(5), 499–509 (2016). 27159850 10.1002/jbio.201500203

[53] Cortes D. H. Jacobs N. T. DeLucca J. F. Elliott D. M., “Elastic, permeability and swelling properties of human intervertebral disc tissues: A benchmark for tissue engineering,” J. Biomech. 47(9), 2088–2094 (2014). 24438768 10.1016/j.jbiomech.2013.12.021 PMC4047194

[54] De S. K. Aluru N. R. Johnson B. Crone W. C. Beebe D. J. Moore J., “Equilibrium swelling and kinetics of pH-responsive hydrogels: Models, experiments, and simulations,” J. Microelectromech. Syst. 11, 544–555 (2002).

[55] Zhang Y. Dong B. Bai Y. Ye S. Lei Z. Zhou Y., “Measurement of depth-resolved thermal deformation distribution using phase-contrast spectral optical coherence tomography,” Opt. Express 23(21), 28067–28075 (2015). 26480464 10.1364/OE.23.028067

[56] Lawman S. Zhang J. Williams B. M. Zheng Y. Shen Y., “Applications of optical coherence tomography in the non-contact assessment of automotive paints,” in *Proceedings of SPIE 103290, Optical Measurement Systems for Industrial Inspection X*, 2017), 103290J.

[57] Haindl R. Trasischker W. Wartak A. Baumann B. Pircher M. Hitzenberger C. K., “Total retinal blood flow and reproducibility evaluation by three beam optical Doppler tomography,” in 26th Conference on Ophthalmic Technologies, Proceedings of SPIE 2016)

[58] Lombardo M. Lombardo G. Carbone G. De Santo M. P. Barberi R. Serrao S., “Biomechanics of the anterior human corneal tissue investigated with atomic force microscopy,” Invest. Ophthalmol. Vis. Sci. 53(2), 1050–1057 (2012). 22266511 10.1167/iovs.11-8720

[59] Fatt I. Goldstick T. K., “Dynamics of water transport in swelling membranes,” J. Colloid Sci. 20(9), 962–989 (1965). 5840305 10.1016/0095-8522(65)90068-1

